# Using Conversations, Listening and Leadership to Support Staff Wellness: The CALM Framework

**DOI:** 10.3390/ijerph22101558

**Published:** 2025-10-13

**Authors:** Usman Iqbal, Natalie Wilson, Robyn Taylor, Louise Smith, Friedbert Kohler

**Affiliations:** 1 Institute for Evidence-Based Healthcare, Faculty of Health Sciences & Medicine, Bond University, Gold Coast, QLD 4226, Australia; 2 Evidence-Based Practice Professorial Unit (EBPPU), Gold Coast Hospital & Health Service (GCHHS), Gold Coast, QLD 4215, Australia; 3 School of Population Health, University of New South Wales, Sydney, NSW 2033, Australia; natalie.wilson1@health.nsw.gov.au (N.W.);; 4 UNSW Clinical Office, Liverpool Hospital (SWSLHD), Sydney, NSW 2170, Australia; 5 Public Health Unit, South Western Sydney Local Health District, Locked Bag 7279, Liverpool, NSW 1871, Australia; 6 School of Clinical Medicine, University of New South Wales, Sydney, NSW 2052, Australia

**Keywords:** healthcare worker, staff wellness rounding, CALM framework, leadership engagement, psychological safety, healthcare leadership, employee well-being, COVID-19

## Abstract

Healthcare workers’ (HCWs) wellness is a critical concern, particularly following the COVID-19 pandemic. Staff Wellness Rounding (SWR) has emerged as a leadership-driven strategy to support HCWs but research on its effectiveness remains limited. This study examines the impact of SWR within a large healthcare organisation in Australia and introduces the CALM (Conversation, Active Listening, Leadership Engagement, Mechanism for Feedback) Framework to enhance leadership-driven wellness initiatives. SWR was implemented across six acute hospitals and 14 community health centres in New South Wales, Australia (July to October 2021). A sequential mixed-methods design was used to evaluate SWR effectiveness, leadership engagement, and key components for a structured wellness approach. Phase One included a survey of 169 HCWs to capture their experiences, and Phase Two and Three comprised semi-structured interviews with SWR leaders, participants of SWR and analysis of 342 SWR records. Findings showed that informal conversations foster trust, active listening supports emotional well-being, and leadership engagement facilitates issue escalation. However, feedback mechanisms require improvement: 77.5% of HCWs felt able to escalate concerns but only 32.5% believed feedback was effectively addressed. These insights directly informed the development of the CALM Framework with implications for leadership training and digital wellness integration in healthcare settings.

## 1. Introduction

The wellness of healthcare workers (HCWs) has become a critical issue, particularly in light of the COVID-19 pandemic, which revealed the vulnerability of HCWs to stressors such as high patient loads, exposure to trauma, long working hours, and emotional strain [1,2]. These stressors contribute to burnout and mental health challenges, which not only affect the workers but also compromise the quality of care delivered to patients [3]. Research demonstrated that HCWs experienced significantly higher levels of emotional distress than the general population during the pandemic, further emphasising the need for structured mental health interventions [1].

To address these concerns, healthcare organisations have implemented various strategies, including leadership-driven wellness interventions like Staff Wellness Rounding (SWR). SWR offers real-time opportunities for HCWs to voice concerns, seek emotional support and connect with resources, helping to mitigate the long-term psychological consequences of crises [4,5]. These rounds also improve communication, build psychological safety and foster team cohesion and morale, especially when led by healthcare leaders [6,7]. Leadership involvement has been shown to improve organisational culture and staff morale, underscoring the importance of leadership in supporting HCW wellness [8]. This is particularly critical in the wake of the COVID-19 pandemic, where frontline healthcare workers faced significant mental health challenges [1,2,9].

Wellness and wellbeing are often used interchangeably in research. The term wellbeing refers to a broader concept including physical, mental and social aspects of wellbeing [10], whereas the term wellness relates to activities, interventions or actions which are more apt for a workplace intervention such as SWR [11].

Despite the growing recognition of leader rounding as an effective intervention to support staff wellness, there is limited research on the role of healthcare leaders without training in psychology or counselling in facilitating wellness rounds [12]. Traditionally, mental health professionals or chaplains have provided emotional support, but leadership-driven conversational approaches in SWR remain underexplored [6]. Leader-staff interactions during SWR can foster psychological safety, enhance leader visibility, and build trust within healthcare teams [13]. A primary barrier to leadership-driven SWR is the reluctance of some leaders to engage in wellness rounding due to a lack of psychological training, a concern highlighted in previous literature [3,14]. Structured frameworks guiding leaders in facilitating meaningful, supportive conversations could strengthen the impact of SWR and ensure alignment with HCWs’ evolving needs during crises [7,15].

This paper proposes the CALM (Conversation, Active Listening, Leadership Engagement and Mechanism for Feedback) framework, developed from a multi-stage study described in the following Section 2. It provides an integrated approach to optimise SWR through leadership-driven wellness initiatives and flexible support strategies. The framework aims to enhance HCW wellness and long-term resilience during high-stress periods, while also informing best practices in healthcare crisis management and leadership-driven wellness interventions.

## 2. Methods

### 2.1. Setting and Participants

The study was conducted in a large, complex healthcare organisation consisting of six acute hospitals and 14 community health centres in New South Wales, Australia. These facilities provided a wide range of services, including acute hospital care, community health, mental health and outpatient services. Healthcare workers from both clinical and non-clinical settings participated in the study. Participants were selected based on their engagement in SWR between July and October 2021 and their involvement in leadership roles related to SWR delivery. We aimed to integrate leadership and conversational approaches into a framework that supports HCW wellness, with a focus on exploring the experiences, benefits and barriers encountered in SWR.

### 2.2. Study Design

**Figure 1:**
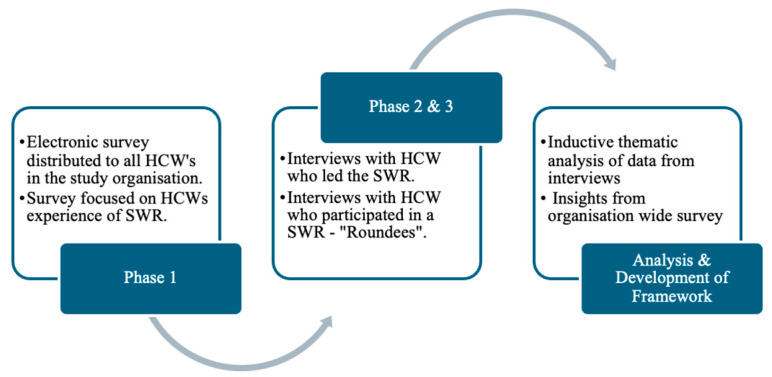
Overview of the CALM study methodology.

The study employed a sequential mixed-methods design, consisting of three phases. Phase 1 involved a survey to gather contextual data on HCW experiences with SWR during the study period (Supplementary Table S1). This phase focused on evaluating the effectiveness and impact of SWR, with particular emphasis on overall satisfaction and perceived benefits. In Phase Two, semi-structured interviews were conducted with SWR leaders to explore the application of SWR, leadership approaches, and challenges encountered in its implementation (Supplementary Table S2). Phase Three of the study included semi-structured interviews with HCW who had participated in a SWR—“roundees”. (Supplementary Table S3). Data from all phases were synthesised to develop the CALM Framework, integrating the most effective leadership strategies and wellness mechanisms identified through the qualitative findings as shown in Figure 1.

### 2.3. Inclusion/Exclusion Criteria

In Phase One, participants were eligible if they had engaged in SWR during the udy period (July–October 2021). In Phase Two, inclusion required participants to have led a minimum of 10 SWR sessions. This was to ensure that the participants in the study had a variety of leader rounding experiences. Those who did not meet these criteria or had left the organisation before completing interviews were excluded. In Phase Three, inclusion required participants to have engaged in a SWR during the study period of July–October 2021.

### 2.4. Data Collection and Analysis

Data were collected through surveys and interviews to provide a comprehensive understanding of the SWR process from both staff and leadership perspectives. An electronic survey was distributed to all HCWs, assessing their overall experience with SWR. The survey included both quantitative (Likert scale) and qualitative questions to allow respondents to share detailed feedback. Semi-structured interviews were conducted with SWR leaders and participants of SWR. The interview questions were designed based on survey findings, aiming to gather in-depth insights into leadership practices, the effectiveness of SWR, and support mechanisms for HCWs.

Quantitative data were analysed using descriptive statistics, while qualitative data from interviews were analysed through inductive thematic analysis using Braun and Clarke’s (2006) method [16]. Two investigators (NW and LS) recorded the interview data via MS Teams and de-identified the data. A third investigator (RT) independently coded the de-identified interview transcripts in NVIVO. Participant responses pertaining to the wellness rounding model’s elements, and its strengths and areas for improvement were collated into themes and sub-themes. This information was extracted from NVIVO into a table noting the theme, sub-theme, researcher interpretation of the theme, and sample participant quotes. That themes table was reviewed by NW, LS, RT and FK. Final themes were agreed upon through discussion and consensus by all investigators.

### 2.5. The Development of the CALM Framework

The CALM framework was developed from the synthesis of key themes identified during the implementation of SWR. The framework integrates four core components: Conversation, Active Listening, Leadership Engagement, and Mechanism for Feedback. This leadership-driven framework was developed to enhance staff wellness by providing a structured approach that integrates leadership principles with conversational techniques in wellness initiatives. It was designed in response to the challenges faced by HCWs during the COVID-19 pandemic, highlighting the need for comprehensive staff support mechanisms.

Through thematic analysis, four core themes were identified: Conversation, Active Listening, Leadership Engagement and Mechanism for Feedback. These were acknowledged as crucial elements of SWR based on both Phase One survey responses and Phase Two interviews with leaders. For example, “Conversation” emerged as a vital aspect due to the unstructured, informal nature of SWR conversations that allowed genuine, candid exchanges. “Active Listening” was identified as a key leadership skill to foster trust, with leaders listening attentively to concerns without rushing to offer solutions. “Leadership Engagement” was found to be central to escalating concerns and providing emotional validation. Lastly, “Mechanism for Feedback” was identified as a critical but underdeveloped aspect, with participants emphasising the need for clearer communication about how feedback is acted upon.

The insights gained from the three phases of the study informed the development of the CALM Framework. Its adaptability as a key feature allows SWR sessions to be tailored to the specific needs of different healthcare areas and staff schedules.

### 2.6. Ethical Approval

The study received ethical approval through the Low or Negligible Risk Review pathway by the Local Health District Human Research Ethics Committee in New South Wales, Australia (2021/ETH11427).

## 3. Results

The development of the CALM framework—Conversation, Active Listening, Leadership Engagement and Mechanism for Feedback—was informed by a multi-phase study, which included 169 staff who participated in a survey and 21 semi-structured interviews across 16 healthcare units. Insights from both the staff survey (Phase One), leader rounder interviews (Phase Two) and roundee interviews (Phase Three) led to the creation of the CALM framework, designed to enhance staff wellbeing and communication. As shown in Table 1, the key findings from the study align with the four components of the CALM framework, illustrating how each element contributes to the wellness rounding process.

The conversational aspect of wellness rounding emerged as a key factor in fostering open communication. Leader rounders required skills that supported open conversations. Roundees valued the informal, unstructured nature of these conversations, which created a safe environment for dialogue. One roundee described it as: “It was really relaxed. It was very informal… It’s not so much like an interview. It’s more like having a coffee with a friend.” This relaxed atmosphere was reinforced in interviews where participants emphasised the openness, noting, “It felt like an openness… an ability to start a conversation,” reflecting the trust and candidness the conversations encouraged.

Active listening was another fundamental element of SWR, which helped staff feel supported and valued. Leader Rounders required skills in active listening and the ability to provide emotional support for staff. Many roundees shared their appreciation for having someone who could listen attentively, with one remarking, “It was important to have someone who took the time and could actually listen.” Phase Two and Three interviews highlighted that listening attentively without rushing to offer solutions was essential, with one leader stating, “Not to fix things, but to pass on the feedback.” This approach contributed to creating a supportive environment where staff felt their concerns were truly heard.

Leadership engagement also played a significant role. Both Leader Rounders and Roundees reported that wellness rounds offered an opportunity to escalate issues to senior management. Leader rounders required skills in theming issues and a process to escalate issues to more senior executives. However, there was also feedback suggesting the inclusion of external facilitators in wellness rounding to offer impartiality, as one roundee explained: “I think it’s also very important for you to have wellness rounding with an external person.” This feedback underscores the value of leadership involvement, but also highlights a desire for a balanced, objective perspective.

While feedback was a central component of the SWR model, the study found challenges in closing the feedback loop. While a significant number of respondents felt able to escalate concerns, fewer believed that these issues were adequately addressed. One roundee pointed out, “I think it’s important to highlight that feedback (from roundees) to the departments,” while another suggested, “Regular updates on how feedback is being utilised would show we’re being heard.” This suggests a need for clearer communication on how feedback is acted upon and for transparency in the process.

**Figure 2:**
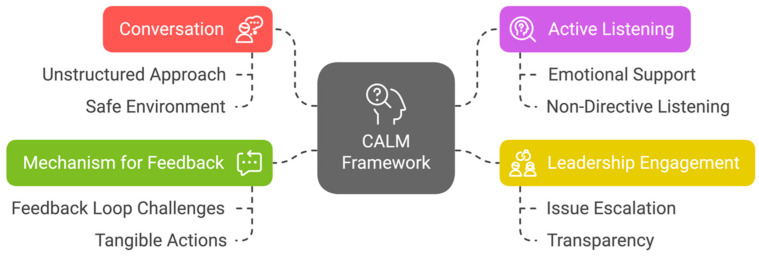
The Conceptual Diagram of the CALM Framework for Staff Wellness Rounding.

The CALM framework, developed based on these findings, aims to foster a supportive environment where healthcare workers feel heard, valued and supported. The conversational and active listening components were especially effective in building trust and emotional connection. Leadership engagement proved vital in facilitating communication and escalating concerns, but challenges remained in ensuring feedback led to visible, actionable changes. The CALM framework offers valuable insights into refining future wellness rounding practices. Figure 2 shows the conceptual diagram of the CALM framework, contextualizing the findings within the broader framework by highlighting the relationships between the four components and their role in enhancing wellness rounding.

## 4. Discussion

The study highlights the essential role of leadership-driven conversational approaches in SWR, particularly through the CALM framework, which ensures that HCWs receive timely, meaningful support. The CALM framework aligns with existing research emphasising the necessity of real-time wellness interventions and structured leadership engagement to mitigate burnout and distress among HCWs [3,6,17,18,19]. This need became especially apparent following the COVID-19 pandemic, which exposed significant mental health challenges among frontline healthcare workers [1,2,9,20]. Despite the benefits observed, challenges remain in implementing leadership-driven SWR, including the reluctance of some leaders to engage in wellness rounding due to a lack of psychological training. These findings align with previous literature on the preparedness of non-clinical leaders to address emotional distress among staff [3,14]. The CALM framework addresses this gap by equipping leaders with structured strategies for navigating sensitive conversations, ensuring that wellness interventions are both feasible and sustainable within healthcare settings [21,22]. Specifically, the Conversation and Active Listening components provide the structured guidance needed for leaders to engage effectively, mitigating their reluctance due to a lack of psychological training.

A key significance of this study is the emphasis on how leader-driven SWR fosters psychological safety, which enhances teamwork, resilience and workplace well-being. Psychological safety, defined as the ability to speak up without fear of negative consequences, is crucial in healthcare settings [5,15,23]. The CALM framework supports this concept by providing healthcare leaders with tools to prioritise active listening and emotional validation, fostering an environment of trust [24]. This is particularly important given that HCWs often face high stress, fatigue and difficulty accessing formal mental health support during crises [9]. Moreover, compromised psychological safety is linked to reduced patient safety and care quality, reinforcing the need for structured leadership engagement in wellness interventions [25].

Informal conversations also play a vital role in strengthening team cohesion and psychological safety. In Study Phase One, 74% of respondents identified informal discussions as valuable, which was further supported by qualitative data from Study 3 where roundees emphasised the need for relaxed and supportive dialogues. Findings from the implementation of an Employee Health and Wellness Programme evaluated using the Reach, Effectiveness, Adoption, Implementation and Maintenance (RE-AIM) framework confirm the value of structured wellness interventions in sustaining wellness and engagement within healthcare settings [26,27]. While formal discussions enhance team dynamics, time constraints and operational demands often limit these interactions. Encouraging more informal conversations through initiatives such as team bonding sessions could strengthen team cohesion and benefit both staff wellness and patient care.

Active listening was identified as a core skill for SWR leaders and emerged as a critical component of effective SWR in this study. Research suggests that structured and empathetic leader-staff interactions improve job satisfaction and reduce stress [13]. The CALM framework operationalises active listening by equipping healthcare leaders with strategies that ensure meaningful dialogue, beyond surface-level check-ins. This structured approach aligns with evidence-based interventions such as the Stability, Encompassing, Endurance and Direction (SEED) wellness model, the Social Support, Tracking Distress, Education And Discussion, communitY (STEADY) wellness programme and RE-AIM, which emphasise the importance of intentional communication in reducing HCW distress [11,26,28]. Similarly, Adibe et al. (2021) developed a proactive wellness model at Rush University Medical Centre to support staff wellness during the COVID-19 pandemic, thereby reinforcing the value of structured, supportive leadership interventions [29].

Leadership engagement, which includes escalation of issues and transparency, is crucial in fostering a responsive organisational culture but it remains inconsistent. While 77.5% of staff felt able to escalate concerns, feedback action was noted as an area for improvement. Roundees expressed a desire for more visible and transparent leadership actions to reinforce trust in the escalation process. These findings highlight the importance of leadership follow-through in building trust. The perceived gap between feedback provision and action implementation is significant, as when employees feel their concerns are not addressed, organisational commitment can decline [26]. Strengthening transparency in leadership decision-making and follow-up communications can enhance trust and accountability. This transparency is also critical for Leader rounders who had reported that they often were not aware of how or if feedback was actioned by management. A process that supports transparency of decision-making across all HCWs would strengthen the SWR model.

In addition, accessibility and logistical barriers, particularly in high-acuity healthcare settings, remain a challenge. These obstacles support findings from studies on staff wellness hubs, where well-designed interventions faced limitations due to time constraints and staff availability [30]. Future research should explore the integration of digital platforms and hybrid wellness rounding frameworks to enhance accessibility and ensure all HCWs, including those in high-pressure environments, benefit from leadership-driven support.

The CALM framework incorporates the feedback mechanism component, enhancing the comprehensiveness of the framework. Only 32.5% of staff reported that feedback was adequately addressed, with structural barriers such as hierarchical organisational structures and time constraints compounding this issue. Effective feedback systems are essential for continuous improvement and staff engagement but are often underdeveloped in healthcare settings [31]. Implementing structured feedback loops with clear action points and accountability measures can improve trust in the system. Also, integrating real-time feedback mechanisms, such as digital platforms for anonymous suggestions, could enhance the effectiveness of feedback processes. Findings from Vanhaecht et al. (2021) suggest that insufficient mental health support systems contribute to worsening psychological wellness among HCWs, highlighting the urgency of structured feedback and intervention systems [9].

### 4.1. Implications for Healthcare Organisations

The CALM approach aligns with organisational priorities such as workforce engagement, improved patient outcomes and a culture of transparency. Each component Conversation, Active Listening, Leadership Engagement and Mechanisms for Feedback contributes uniquely to these objectives. However, their maximum impact can only be realised through systemic integration. Healthcare institutions that prioritise communication and responsiveness see measurable improvements in staff satisfaction and retention. Embedding CALM concepts into leadership models and training programmes can create a more resilient and responsive healthcare workforce [32]. While the CALM framework offers a comprehensive structure for improving workplace communication, further research is needed to evaluate its long-term impact. Future studies could explore the effectiveness of targeted interventions, such as leadership development programmes and structured conversation initiatives, in reinforcing CALM values [5]. Furthermore, cross-institutional comparisons could provide valuable insights into best practices and scalable solutions for healthcare settings. A key area for future investigation is the integration of digital tools to facilitate real-time feedback and communication. Digital wellness initiatives can enhance accessibility and engagement, reinforcing the potential for technology-driven approaches in supporting HCW wellness [29]. Emerging technologies such as AI-driven sentiment analysis and feedback platforms could further refine the CALM framework’s application across diverse healthcare contexts.

### 4.2. Future Directions

The findings emphasise the importance of healthcare organisations prioritising leadership training in emotional intelligence, active listening, and crisis communication. Embedding these skills into leadership development programmes can enhance the effectiveness of SWR and other wellness initiatives. In addition, incorporating structured feedback mechanisms such as real-time staff input and longitudinal assessments can ensure continuous improvement in wellness rounding practices while supporting long-term organisational commitment to HCW mental health support [4,9]. Moreover, healthcare organisations must recognise the need for flexibility and adaptability in leadership-driven wellness initiatives. As demonstrated by the SEED wellness model, the STEADY wellness programme and RE-AIM, organisational flexibility is key to addressing the evolving needs of HCWs, particularly during crises [11,28]. These models, like the CALM framework, also demonstrate the importance of intentional and flexible approaches to adapt to the evolving needs of healthcare workers during crises. The CALM framework provides a structure for integrating flexibility, ensuring wellness initiatives remain relevant and responsive to HCWs’ needs.

### 4.3. Strengths and Limitations

This study offers valuable insights into the CALM framework’s application in leadership-driven wellness rounding within a large healthcare organisation. A key strength lies in its sequential mixed-methods design, which integrates both quantitative survey data and qualitative interview findings to provide a comprehensive understanding of the challenges and benefits of SWR. The inclusion of real-world implementation data enhances the ecological validity of the findings, making them highly relevant to contemporary healthcare settings. Also, the study’s emphasis on leadership engagement, active listening and structured feedback mechanisms highlights practical strategies for mitigating HCW burnout and stress, offering actionable insights for improving workplace well-being.

However, some limitations should be noted. While the study was conducted across multiple acute hospitals and community health centres, the findings may not be fully generalisable to other healthcare systems with different organisational structures or cultural contexts. The qualitative data, while rich in detail, may be subject to interpretation bias and self-reported survey responses could introduce response bias, where participants may have reported more favourable experiences than those observed in practice.

The study data related to the experience of HCW during the COVID-19 pandemic. The unique experience of living and working in the COVID pandemic environment may have impacted the findings of this study and may not be replicated if a different pandemic or crisis occurred.

Furthermore, as the CALM framework was developed through this study, its long-term impact, outcomes and sustainability remain unknown. Future research should include longitudinal studies to assess the lasting outcomes of CALM-based interventions and explore how tools can enhance real-time feedback and communication processes.

## 5. Conclusions

The CALM framework enhances healthcare staff wellness by fostering leadership-driven conversations, prioritising active listening, and promoting psychological safety. Through strengthening team cohesion, trust and communication, the framework aligns with key organisational goals and supports long-term resilience. Further optimisation through leadership training, structured feedback systems and digital tools will ensure a more responsive and supportive healthcare environment.

## Figures and Tables

**Table 1:** Summary of Findings and Alignment with CALM Framework Components for Staff Wellness Rounding.

CALM Framework Component	Findings from Staff Survey (Phase One)	Findings from Leader Rounder Interviews (Phase Two)	Findings from Roundee Interviews (Phase Three)	Key Observations
Conversation	74% valued informal, open conversations. 85.2% liked positive reinforcement.	Leader Rounders required skills that support staff to have open conversations	Roundees appreciated the relaxed, non-judgmental atmosphere.	Informal conversations foster trust and openness, building connection.
Active Listening	74% felt heard, and desired compassionate listening rather than immediate solutions.	Leader rounders required active listening skills, and the ability to provide emotional support	Roundees valued active listening and emotional support.	Active listening and emotional support are essential, with a preference for listening over problem-solving.
Leadership Engagement	77.5% felt wellness rounds allowed them to escalate issues to management.	Leaders acted as communicators and facilitators of staff feedback	Leaders acted as communicators and facilitators for staff feedback.	Leadership plays a pivotal role in feedback escalation, addressing concerns, and supporting staff.
Mechanism for Feedback	77.5% felt they could escalate issues, but only 32.5% felt feedback was adequately addressed.	Leader rounders required a process and the skills to theme feedback and to escalate issues to senior executive	Roundees wanted more visible actions on their feedback.	A gap exists between feedback collection and tangible change, indicating a need for greater transparency and accountability.

## Data Availability

The contributions presented in this study are included in the article/Supplementary Material. Further inquiries can be directed to the corresponding author.

## Supplementary Materials

The following supporting information can be downloaded at: https://www.mdpi.com/article/10.3390/ijerph22101558/s1, Table S1: Staff Wellness Rounding—Survey Question Set; Table S2: Semi structured Interviews for the leaders who did the Staff Wellness Rounding; Table S3: Semi-structured Interview Questions for Roundees.

## Abbreviations

The following abbreviations are used in this manuscript:

CALM	Conversation, Active Listening, Leadership Engagement, Mechanism for Feed-back
SWR	Staff Wellness Rounding
HCWs	Healthcare workers
