# Supplementary material for: Using Conversations, Listening and Leadership to Support Staff Wellness: The CALM Framework
Source: Int J Environ Res Public Health. 2025 Oct 13;22(10):1558. doi: 10.3390/ijerph22101558 (PMC12563315; doi:10.3390/ijerph22101558)
Supplement: Supplementary file 1 [file ijerph-22-01558-s001.zip › Supplementary File S1- SWR Survey question set.pdf]

Table S1: Staff Wellness Rounding – Survey Question Set

	Question	Answers
1	Have you been involved in staff wellness rounding in the last 6 months?	Yes→ Move to question 2.<br>No → End survey<br>Unsure → End survey
2	Who did the staff wellness rounding with you or your team?	Your Manager/Leader<br>A member of the Staff Wellness Team<br>Unsure
3	How was the staff wellness rounding delivered?	Face to face<br>Virtual (Skype, MS Teams, Zoom)<br>Phone Call<br>Unsure
4	What was the format of the staff wellness round?	One to one (individual session)<br>Group Session
5	Did staff wellness rounding provide an opportunity for you to escalate issues or concerns you have related to COVID_19	Strongly agree.<br>Agree<br>Neither agree nor disagree<br>Disagree<br>Strongly disagree
6.	Did staff wellness rounding provide an opportunity to recognise or acknowledge staff member(s) for their work?	Strongly agree.<br>Agree<br>Neither agree nor disagree<br>Disagree<br>Strongly disagree
7	Did staff wellness rounding provide guidance about how to access further support and or information about staff wellness?	Strongly agree.<br>Agree<br>Neither agree nor disagree<br>Disagree<br>Strongly disagree
8	I felt that my concerns were listened to and heard during staff wellness rounding.	Strongly agree.<br>Agree<br>Neither agree nor disagree<br>Disagree<br>Strongly disagree
9	I felt safe to raise my concerns during staff wellness rounding?	Strongly agree.<br>Agree<br>Neither agree nor disagree<br>Disagree<br>Strongly disagree
10	Would you recommend staff wellness rounding to other staff?	Yes<br>No<br>Unsure
11	Why or why not?	Free Text
12	Did you feel like anything changed because of wellbeing ie issues were addressed or initiatives implemented?	Yes<br>No<br>Unsure
13	Do you have any comments about SWR ?	Free text
14	Please select the Facility or Service that you primarily work at from the list below	XX
15	Please select your work place discipline from the list below	XX
