# Supplementary material for: Using Conversations, Listening and Leadership to Support Staff Wellness: The CALM Framework
Source: Int J Environ Res Public Health. 2025 Oct 13;22(10):1558. doi: 10.3390/ijerph22101558 (PMC12563315; doi:10.3390/ijerph22101558)
Supplement: Supplementary file 1 [file ijerph-22-01558-s001.zip › Supplementary File S3 - Semi-structured Interview Questions.pdf]

Table S3: Semi-structured Interview Questions for Roundees

Thank you for agreeing to participate in this interview. This will take approximately 30 -40 minutes. Approval has been received from ethics for approval as part of Phase 2 of this study. Please ensure that you have received and read the Participant information sheet to complete the consent prior to completion of this interview. If you have completed these, are you happy to continue?

Objectives

Understand and evaluate Staff Wellness Rounding (SWR) in XX. This includes:

- Understanding the effectiveness of SWR for managers and frontline staff,
- Determining the impact of SWR in the organisation
- Evaluating the current model of SWR

The following questions have been developed as a part of a semi-structured interviews based on the feedback from a staff survey in order to attain a deeper understanding of the perspectives of the rounders and roundees.

Question Set

Themes	Questions for roundees
**Preparedness/Engagement**	In your words, what was the intention of the wellness rounds?<br>When did you encounter the wellness round?<br>E.g. time of day vs crisis period or time of need such as covid<br>When do you feel wellness rounds should be conducted?<br>Who should conduct the wellness rounds?<br>Prompt 1 independent/external vs manager/executive vs peers<br>Describe what the pros and cons would be?<br>Did you feel comfortable talking to this person and why?<br>Did you feel the rounder clearly described the intention of the round<br>Can you describe this in your own words?<br>How did you prepared to engage in wellness rounds?
	Who do you think is the best person to do wellness rounds?
**Actions**	Did you feel like you were able to raise issues?<br>Did you feel like your issues were heard?<br>What made you feel able to raise issues or be heard?<br>Were your issues/concerns escalated or addressed?
**Feedback**	Did you receive feedback about wellness issues raised?<br>Did you feel the rounds addressed staff wellbeing as a result?<br>How did you feel the rounder received your feedback? (reception of the rounder)<br>How would you like to receive feedback regarding the actions in the future?
**General evaluation**	What did you like about the wellness rounding?<br>What could be improved?
**Impact**	How did you feel the rounds impacted on the roundee (staff) wellness or wellbeing?
