# Supplementary material for: Using Conversations, Listening and Leadership to Support Staff Wellness: The CALM Framework
Source: Int J Environ Res Public Health. 2025 Oct 13;22(10):1558. doi: 10.3390/ijerph22101558 (PMC12563315; doi:10.3390/ijerph22101558)
Supplement: Supplementary file 1 [file ijerph-22-01558-s001.zip › Supplementray FIle S2 -SWR Interview Question Set.pdf]

**Table S2: Semi structured Interviews for the leaders who did the Staff Wellness Rounding.**

**Script:**

Thank you for agreeing to participate in this interview. This will take approximately 30 -40 minutes. Approval has been received from ethics for approval as part of Phase 2 of this study. Please ensure that you have received and read the Participant information sheet to complete the consent prior to completion of this interview. If you have completed these, are you happy to continue?

Based on feedback from the survey that was distributed to staff and managers, it became apparent that to meet our objectives of the research, we needed to interview those who participated in rounding (roundees) and those who rounded with staff (rounders).

**Objectives**

- Understand and evaluate Staff Wellness Rounding (SWR) in XX. This includes:
    - understanding the effectiveness of SWR for managers and frontline staff,
    - determining the impact of SWR in the organisation
    - evaluating the current model of SWR

The following questions have been devised as a part of a semi-structured interview based on the feedback from a staff survey in order to attain a deeper understanding of the perspectives of the rounders and roundees.

**Question Set**

**Understanding of SWR**

- In your words, what was the intention of the SWR?

**When and who should SWR**

- When did you do SWR? Why did you do them at those times?
- How did you engage with staff in SWR eg: face to face, telephone?
- When do you feel SWR should be conducted and by whom?

**Skills and Knowledge**

- Did you feel you were skilled enough to do SWR?
- What preparation and training do you feel a SWR needs?

**Outcomes**

- How do you escalate the outcomes of the SWR?
- How are the outcomes/actions of the SWR communicated back to staff, escalated to leaders?
- How do you determine what gets escalated or addressed from a SWR?

**Other**

- What did you like about SWR?
- What could be improved about SWR?
- How did you feel the SWR impacted on the roundee (staff) wellness or wellbeing?
- Is there anything else you would like to add about SWR?
